# The effect of using genealogy-based haplotypes for genomic prediction

**DOI:** 10.1186/1297-9686-45-5

**Published:** 2013-03-06

**Authors:** Vahid Edriss, Rohan L Fernando, Guosheng Su, Mogens S Lund, Bernt Guldbrandtsen

**Affiliations:** 1 Center for Quantitative Genetics and Genomics, Department of Molecular Biology and Genetics, Aarhus University, Tjele DK-8830, Denmark; 2 Department of Animal Science, Iowa State University, Ames, IA 50011, USA

## Abstract

**Background:**

Genomic prediction uses two sources of information: linkage disequilibrium between markers and quantitative trait loci, and additive genetic relationships between individuals. One way to increase the accuracy of genomic prediction is to capture more linkage disequilibrium by regression on haplotypes instead of regression on individual markers. The aim of this study was to investigate the accuracy of genomic prediction using haplotypes based on local genealogy information.

**Methods:**

A total of 4429 Danish Holstein bulls were genotyped with the 50K SNP chip. Haplotypes were constructed using local genealogical trees. Effects of haplotype covariates were estimated with two types of prediction models: (1) assuming that effects had the same distribution for all haplotype covariates, i.e. the GBLUP method and (2) assuming that a large proportion (π) of the haplotype covariates had zero effect, i.e. a Bayesian mixture method.

**Results:**

About 7.5 times more covariate effects were estimated when fitting haplotypes based on local genealogical trees compared to fitting individuals markers. Genealogy-based haplotype clustering slightly increased the accuracy of genomic prediction and, in some cases, decreased the bias of prediction. With the Bayesian method, accuracy of prediction was less sensitive to parameter π when fitting haplotypes compared to fitting markers.

**Conclusions:**

Use of haplotypes based on genealogy can slightly increase the accuracy of genomic prediction. Improved methods to cluster the haplotypes constructed from local genealogy could lead to additional gains in accuracy.

## Background

Genomic prediction is a method that uses genome-wide dense markers to predict additive genetic values [1]. It was originally assumed that the key feature of this method was that markers were in linkage disequilibrium (LD) with the quantitative trait loci (QTL) and explained most of the genetic variance [2]. However, the genetic variance explained by single nucleotide polymorphism (SNP) markers depends also on the additive genetic relationships between individuals. The accuracy of genomic prediction increases when the markers explain more additive genetic relationships between individuals. Previous studies e.g. [3] have reported that accuracy of genomic prediction increases as the genetic relationship between candidates and reference animals increases. Habier et al. [4] showed by simulation that a large part of the accuracy of genomic prediction was due to genetic relationships captured by markers. Recent studies in a sheep population have demonstrated that markers on a single chromosome could capture up to 86% of the accuracy of genomic prediction that was achieved when using all markers [5]. These results support the fact that most of the accuracy of genomic prediction comes from tracing genetic relationships between individuals. Genetic gain from LD information is expected to increase if the disequilibrium between markers and QTL is stronger. An alternative to using individual markers for prediction is to construct haplotypes based on several markers surrounding a QTL. The probability for a QTL to be in strong LD is higher with a haplotype of markers than with an individual marker [2].

A drawback of using haplotypes instead of individual markers in genomic prediction is that many more effects need to be predicted. When the number of covariates increases, the amount of data available for each covariate decreases and consequently the accuracy of the predicted covariate effects is reduced. However, several simulation studies have shown that using haplotypes instead of individual markers increases the accuracy of genomic prediction [6-8]. To date, only a few studies have investigated the use of haplotypes for genomic prediction in real data [9,10]. Boichard et al. [9] focused on SNP haplotypes related to QTL with large or moderate effects in French dairy cattle. De Roos et al. [10] used ancestral haplotypes for genomic prediction in a Holstein population.

Several methods are available to construct marker haplotypes. Some are simple, e.g. grouping SNP based on counts of markers [7] or fixed lengths of chromosome segments. Other methods use more complicated algorithms to group SNP and cluster the resulting haplotypes. Calus et al. [6,8] used an identity by descent (IBD) matrix to group markers and construct haplotypes, and then applied different IBD probability thresholds to cluster similar haplotypes. In this method, the number of haplotype effects to be estimated depended on the IBD probability threshold and on the number of markers included in each haplotype. A lower IBD threshold and more markers per haplotype reduced the number of haplotype effects to be predicted. Here, we used local genealogies to construct haplotypes and to define haplotype clusters.

At each point in the genome, extant haplotypes are related in a genealogical tree that ultimately leads back to a common ancestor. Any mutations that are currently segregating in the population necessarily must have occurred at the position of the mutation at some point in the local genealogy, i.e. it must have happened on an edge of a local genealogy [11]. Given perfect reconstruction of the local genealogy, haplotypes that carry alternative causative polymorphisms will be perfectly clustered by splitting at the edge where the mutation occurred. Thus, for a bi-allelic causative polymorphism, local genealogy haplotype clustering should yield the optimal clusters of haplotypes. However, whether this clustering is optimal or not, also depends strongly on the accuracy of the reconstructed local genealogies.

Two classes of prediction models have been used to estimate haplotype effects. One class of models assumes that effects of all haplotypes have the same distribution, e.g. GBLUP or random regression BLUP (RR-BLUP). The other class of models are mixture models, like BayesB or BayesC, which assume that a large proportion of haplotypes have zero effect, while a small proportion have non-zero effects. These models select the most informative haplotypes for genomic prediction and capture LD information better than the GBLUP models [4].

The aim of this study was to investigate the accuracy and bias of genomic prediction using haplotypes based on local genealogy information in a Danish Holstein cattle population, applying GBLUP, BayesB and BayesC prediction models.

## Methods

### Data

A mixture of versions 1 and 2 of the Illumina Bovine SNP50 BeadChip [12] was used to genotype 4429 Danish Holstein bulls born between 1974 and 2006. Data for SNP with a minor allele frequency less than 0.01, with no valid chromosome position in the UMD3.1 assembly [13], an average GC score less than 0.15, and SNP on the sex chromosomes were removed from the dataset. After editing, 43 503 markers remained across 29 autosomes.

Response variables in the genomic prediction models were deregressed EBV [14,15]. Detailed descriptions of deregressed EBV (DRE) for three index traits, fertility, protein yield and mastitis, are provided in Table 1. More information on the EBV of index traits is available from the Danish Cattle federation [16]. Reliabilities of the DRE $\left(r_{DRE}^{2}\right)$depend on the heritability (h^2^) of the trait and the effective daughter contribution (EDC) of individuals and was calculated as $r_{DRE}^{2}=\text{EDC}/\left(\text{EDC},+,k\right)$, where k = (4 - h^2^)/h^2^.

**Table 1:** Data for three traits with reference and test datasets

**Trait**	**h**^**2**^	**Reference**				**Test**			
		**n**	$r_{DRE}^{2}$	**range (DRE)**	**median (DRE)**	**n**	$r_{DRE}^{2}$	**range (DRE)**	**median (DRE)**
Fertility	0.04	3084	0.67	7.8-251.7	105.4	1267	0.58	19.1-202.2	103.7
Protein	0.39	3040	0.94	49.0-145.3	92.5	1292	0.92	59.6-153.8	106.0
Mastitis	0.04	3081	0.76	44.6-165.7	101.1	1333	0.67	43.3-188.6	103.4

h^2^: heritability; n: number of bulls; $r_{DRE}^{2}$: average reliabilities; range (DRE) and median (DRE): range and median of de-regressed EBV (DRE) for bulls; Reference: reference dataset; Test: test dataset.

### Genomic prediction

Marker and haplotype-based predictions were performed and their results were compared. Haplotype-based predictions used genealogically related haplotypes that were clustered together. Genealogical relationships between haplotypes were estimated based on local genealogies.

### Local genealogy haplotype clustering

Marker data were phased and imputed with Beagle 3.3 [17]. First, Beagle constructed haplotypes with default parameter values for scale = 4.0 and shift = 0.2. Then, conditional on the inferred haplotypes, all missing genotypes were imputed using Beagle’s hidden Markov model. After obtaining the phased and imputed data for each individual, local genealogical trees were constructed using the Blossoc software [18]. In Blossoc, a genealogy is represented as a single rooted binary tree topology, which is constructed around each marker. The procedure was as follows: first, the four-gamete rule was used to find the largest segment around each marker that does not require recombination to be inferred. Assuming an infinite sites model, if all the four possible haplotypes of two markers are observed (00, 01, 10 and 11), a single genealogy that does not incorporate recombination can be reconstructed [18]. For each marker, Blossoc considers the region around that marker that includes as many markers as possible without violation of the four-gamete rule. Then, an unrooted genealogy was reconstructed for this region. The root of this local genealogy tree was on the branch of the tree where the mutation for the current marker occurred. An example of construction of the tree and graphical representations of the tree can be found in [18] and [11].

Haplotypes were clustered based on the reconstructed genealogy. Local genealogical trees contain many levels but only the first three levels were considered, because as the number of levels increases, the frequency number of haplotypes per cluster decreases and this could lead to numerical instability. The first level consisted of the root marker. The second level had two nodes and the third level four nodes. Each node had two branches and every branch below the third level was considered as one cluster. After constructing the tree, there was a maximum of eight clusters of haplotypes per tree. This process was repeated for all markers in the dataset.

Each individual had two haplotypes (one maternal and one paternal) in each genealogy and each haplotype belonged to exactly one of the eight clusters. Thus, each individual possessed 0, 1 or 2 copies of each haplotype. In haplotype-based prediction, these numbers replace the marker allele counts in genomic prediction.

### Statistical models

One non-Bayesian method, i.e. GBLUP and four Bayesian methods, i.e. BayesB, BayesBπ, BayesC and BayesCπ, were used to predict direct genomic values (DGV). Prediction was done using individual markers or haplotypes as covariates and resulting predictions were compared.

### Bayesian methods

In BayesB [1], each covariate had its own variance with a scaled inverse chi-square prior [19]. The proportion of covariates with zero effect, π, was set to 0.99. BayesC also fitted data using a mixture distribution of marker effects, with effects equal to zero with probability π but an effect that is sampled from a normal distribution with mean zero and a variance parameter that is shared for all markers with non-zero effects with probability 1-π. This common variance is treated as unknown and has a scaled inverse-chi square prior with 4.2 degrees of freedom and the same scale as derived for BayesB. More details are available in [19]. For comparison, BayesC was fitted with π fixed at 0.99 and 0.999.

BayesCπ is similar to BayesC. However, in contrast to BayesC, π was treated as an unknown parameter with a uniform (0,1) prior distribution. The actual value was sampled conditional on the data as part of the Gibbs sampler. The posterior mean of π from BayesCπ was also used as the fixed value in BayesB, which will be referred to as BayesBπ.

The general Bayesian statistical model was:

$$y=1\mu +\sum_{i=1}^{K}z_{i}a_{i}+e,$$

where **y** is the vector of DRE, **1** a vector of 1^′^s, *μ* the overall mean, *K* the number of covariates, **z**_*i*_ an N × 1 vector of genotypes at SNP *i* with individual marker-based prediction and the number of haplotype copies for each animal in covariate *i* with haplotype-based prediction, a_*i*_ a covariate effect *i*, with $a_{i}∼N\left(0,\sigma_{a}^{2}\right)$ (with probability 1 - π) or a_*i*_ = 0 (with probability π), **e** a vector of residual effects and $e∼N\left(0,{D\sigma }_{e}^{2}\right)$. **D** is a diagonal matrix with element d_ii_ = 1/w_i_, where w_i_ is a weighting factor for the i^th^ DRE. The weighting factor, w_i_ = reliability of DRE_i_/(1-reliability of DRE_i_), was applied to account for heterogeneous residual variances due to different reliabilities of DRE. To avoid possible problems caused by extremely high weights, reliabilities larger than 0.98 were replaced by 0.98 in the calculation of weights. Details concerning the estimation of $\sigma_{a}^{2}$ are described in Habier et al. [18].

All analyses were carried out using the GenSel software [20]. The Gibbs sampler was run as a single chain with a length of 10 000 samples, of which the first 1000 samples were discarded as burn-in. DGV were estimated as the posterior mean of the sum of a_*i*_ from the remaining 9000 samples. A Gibbs sampler with a longer chain and burn-in (50 000 samples and 20 000 as burn-in) was tested and produced the same results. Convergence of estimated parameters was visually examined and a chain of 10 000 samples was found to be sufficient.

### GBLUP analysis

The GBLUP model [21,22] was:

y=1μ+Zg+e,

where **y** is the vector of DRE, **1** a vector of 1^′^s, *μ* the overall mean, **Z** the design matrix of SNP genotypes or haplotypes covariates associating **g** with response variables, **g** the vector of covariate effects with $g∼N(0,{G\sigma }_{g}^{2})$, where $\sigma_{g}^{2}$ is the additive genetic variance, **G** the realized genomic relationship matrix and **e** the vector of random residuals with $e∼N\left(0,{D\sigma }_{e}^{2}\right)$. **D** was the same as in the Bayesian method. Details of the model and construction of the **G** matrix are in [23]. When using haplotypes to construct **G**, the matrix (**M**) that links haplotypes to individuals and its number of columns is equal to the number of haplotype covariates. The element in row *i* and column *j* of this matrix was equal to 0, 1 or 2, corresponding to the number of copies of haplotypes *j* in individual *i*. Then, matrix **G** was calculated in the same way as with use of marker genotypes. Based on the present data, the two **G** matrices built using either marker genotypes or haplotypes were very similar. The correlation coefficient between the two **G** matrices was 0.995 for the diagonal elements and 0.944 for the off-diagonal elements. The GBLUP analyses were performed using the DMU package [24].

### Validation of genomic predictions

The predictive ability of each model was assessed using a validation procedure in which the whole dataset was divided into two parts: 3084 Holstein animals born before October 1 2001 constituted the reference population and 1333 animals born after that date, the test population. Because not all genotyped animals had DRE for all three traits, the number of animals in the reference and test datasets differed between traits (Table 1). Accuracies of genomic predictions were measured as the correlation between DRE and DGV divided by the square root of the average reliability of DRE for animals in the test population [23]. Bias of genomic predictions was assessed by examining the regression of DRE on DGV in the test population. The standard errors of the regression coefficients and accuracies were calculated using R (v. 2.15) (http://www.r-project.org/).

## Results

After editing, the dataset contained 43 503 markers. Construction and cutting of local genealogies produced 326 055 haplotype covariates. The total number of covariates divided by the total number of markers was around 7.5, i.e. less than 8 covariates per marker, since some local genealogies were very unbalanced with some branches empty. Not all markers had a complete tree. A tree was complete when all the nodes in the three levels had two branches such that there were eight clusters of haplotypes in the bottom of the tree to use for prediction.

Accuracies of DGV and their standard errors in the test population using different prediction methods with individual markers and haplotypes as predictors are shown in Table 2, along with the posterior mean of π from the BayesCπ method. For all three traits, the lowest accuracies were obtained for BayesC when π was fixed at 0.999 for both individual marker-based and haplotype-based prediction. The prediction method yielding the highest accuracy differed between traits: BayesBπ had the highest accuracy for protein yield, BayesCπ for mastitis and GBLUP for fertility when individual marker prediction was used. The number of covariates was larger with haplotype-based prediction than with individual marker-based prediction. Except for the BayesC model with π = 0.999, accuracies from different prediction methods using haplotypes were similar but the highest accuracies were obtained for different traits with different methods i.e. for protein yield with BayesCπ, for mastitis with BayesBπ and for fertility with BayesC and π = 0.99. With both individual marker-based and haplotype-based prediction, BayesCπ had the highest or close to the highest accuracy for all traits. The posterior means of π were similar with individual marker-based and haplotype-based predictions for mastitis and fertility, but increased from 0.91 to 0.97 with haplotype-based prediction for protein yield.

**Table 2:** Accuracy of genomic predictions (ACC) and standard errors (SE) for three traits

**Prediction method**	**Fertility**		**Protein yield**		**Mastitis**	
	**ACC**	**SE**	**ACC**	**SE**	**ACC**	**SE**
**Individual marker prediction**						
GBLUP	0.599	0.013	0.646	0.016	0.622	0.013
BayesC π = 0.999	0.521	0.024	0.558	0.023	0.526	0.023
BayesC π = 0.99	0.574	0.023	0.625	0.022	0.595	0.022
BayesCπ	0.596	0.023	0.650	0.021	0.629	0.021
**π**	**0.91**		**0.91**		**0.90**	
BayesBπ	0.594	0.023	0.651	0.021	0.623	0.021
BayesB π = 0.99	0.570	0.023	0.624	0.022	0.586	0.022
**Haplotype prediction**						
GBLUP	0.596	0.013	0.651	0.016	0.628	0.013
BayesC π = 0.999	0.566	0.023	0.611	0.022	0.575	0.022
BayesC π = 0.99	0.598	0.023	0.656	0.021	0.629	0.021
BayesCπ	0.596	0.023	0.658	0.021	0.629	0.021
**π**	**0.90**		**0.97**		**0.89**	
BayesBπ	0.593	0.023	0.657	0.021	0.633	0.021
BayesB π = 0.99	0.595	0.023	0.651	0.021	0.624	0.021

The regression coefficients of DRE on DGV and their standard errors for individual marker-based and haplotype-based prediction in the test population are shown in Table 3. All the regression values were less than 1, indicating that the variance of the DGV was over-predicted to some extent. This means that positive values of DGV over-predict DRE and negative DGV values under-predict DRE. BayesC with π = 0.999 had the largest deviation from 1, i.e. the DGV obtained with this model were the most biased. GBLUP led to the lowest bias for protein yield and fertility for both individual marker-based and haplotype-based prediction and the second lowest bias for mastitis. BayesBπ produced the least biased DGV for mastitis. The lowest standard error of the regression coefficient was observed for protein yield, followed by mastitis and fertility. As shown in Table 3, in most cases the regression coefficients were closer to 1 with haplotype-based prediction than with individual marker-based prediction. The only exception was BayesBπ for fertility.

**Table 3:** Regression coefficients (REG) and standard errors (SE) of de-regressed EBV on genomic prediction

**Prediction method**	**Fertility**		**Protein yield**		**Mastitis**	
	**REG**	**SE**	**REG**	**SE**	**REG**	**SE**
**Individual marker prediction**						
GBLUP	0.968	0.053	0.869	0.031	0.969	0.045
BayesC π = 0.999	0.848	0.055	0.744	0.033	0.861	0.050
BayesC π = 0.99	0.915	0.053	0.827	0.031	0.930	0.046
BayesCπ	0.956	0.053	0.869	0.031	0.966	0.044
BayesBπ	0.922	0.051	0.847	0.030	0.978	0.045
BayesB π = 0.99	0.911	0.053	0.825	0.031	0.935	0.047
**Haplotype prediction**						
GBLUP	0.961	0.053	0.891	0.031	0.987	0.045
BayesC π = 0.999	0.877	0.052	0.826	0.032	0.908	0.047
BayesC π = 0.99	0.956	0.052	0.871	0.030	0.972	0.044
BayesCπ	0.956	0.053	0.884	0.030	0.980	0.045
BayesBπ	0.906	0.050	0.850	0.029	0.996	0.045
BayesB π = 0.99	0.926	0.051	0.850	0.030	0.985	0.046

## Discussion

Use of genealogy-based haplotypes instead of individual marker genotypes had an effect on the accuracy of genomic prediction. Simulation studies have shown that using SNP haplotypes improves the accuracy of genomic prediction [6-8]. Different methods and different numbers of markers per haplotype have been used to construct and cluster haplotypes. Although there are many simulation studies on genomic prediction using haplotypes, studies based on real data in dairy cattle are limited. This study investigated the accuracy and bias of DGV derived using local genealogy haplotypes in the Danish Holstein population genotyped with the 50 K SNP chip.

In our study, the haplotype-based prediction approach slightly increased the accuracy of DGV compared to individual marker-based genomic prediction in some cases. The biggest gain in accuracy was achieved for protein yield, followed by mastitis. A previous study reported increased accuracy of DGV using a model with selected haplotypes and polygenic effects in French dairy cattle [9].

There could be several reasons why, in our study, only small increases in the accuracy of DGV were obtained when using haplotype-based prediction. First, local genealogies were used to cluster haplotypes. The true genealogy is unknown and must be inferred. There is a trade-off between accuracy and computing time when inferring the genealogy. The Blossoc method aims at achieving computing efficiency [11]. Overall, it took 15 h to construct genealogy trees for 43 503 markers and 4429 animals with a server with 2.93 GHz CPU and 48 GB RAM. Reconstructing local genealogies more accurately could further increase the accuracy of DGV. Another reason for the small increases in accuracy from using haplotypes could be that the tree was cut at the third level to avoid numerical problem. Cutting the tree at deeper levels (e.g. fourth or fifth level) would produce more haplotype clusters and thus haplotypes could be clustered more accurately. However, this would also reduce the number of individuals with data for each cluster and reduce the accuracy of the estimated effects of the haplotypes. In our study, given the size of the available population, cutting at the third level was assumed to be optimal. In cases with larger populations, cutting at lower levels might be considered.

In general, two sources of information can influence the accuracy of genomic prediction i.e. (1) LD between markers and QTL and (2) genetic relationships between individuals that are captured by markers. The initial assumption was that most of the accuracy of genomic prediction arises from LD [2]. Capturing more LD will increase the accuracy of genomic prediction, which was the main idea of using haplotypes. However, several recent studies suggest that a large part of the accuracy of genomic prediction is derived from reconstruction of genetic relationships rather than from close LD between markers and QTL. Daetwyler et al. [5] using data from a sheep population showed that markers from a single chromosome captured 86% of the accuracy of genomic predictions using all markers on the ovine 50 K SNP chip [5]. These results indicate that there is a small opportunity to improve genomic prediction by capturing more LD information. Thus, constructing haplotypes from SNP markers is not expected to greatly increase the accuracy of genomic prediction.

Bias of genomic predictions was assessed by the regression coefficient of DRE on DGV in the test population (Table 3). Regression coefficients were less than 1 for all traits considered and all models applied. This shows an inflation of genomic predictions. Haplotype-based prediction led to less bias than individual marker-based prediction.

Two classes of prediction models were applied in our study: GBLUP, with the effects of all covariates following the same distribution, and Bayesian methods, in which a high proportion of covariates had zero effect (π) and a small proportion had moderate to large effects. With GBLUP, haplotype-based prediction fitted around 7.5 times more covariate effects but with only a small gain in accuracy for protein and mastitis traits, compared with individual marker-based prediction. With the Bayesian methods, π had a strong effect on the accuracy of genomic prediction when using individual markers. Forcing a higher proportion of covariates (π = 0.99) to have zero effect decreased the accuracy of DGV when using individual markers but had less influence when using haplotypes. In methods like BayesB, π is usually fixed before the analysis and finding the most appropriate π is challenging. With haplotype-based prediction, DGV accuracies were less influenced by the value of π. Thus, the challenge consisting in finding the most appropriate π value can be relaxed when using the haplotype-based approach. The accuracy of haplotype-based prediction decreased when a very high proportion of haplotype covariates were forced to have zero effect (π = 0.999). Loss of accuracy by shrinking a high proportion of the predictor effects to zero indicates that predictors with real predictive power are being removed from the model.

For protein yield, using the Bayesian haplotype-based model led to a larger posterior mean of the fraction (π) of predictors whose effects were set to 0, compared to the individual marker-based model. This was not the case for fertility or mastitis. Assuming that the major source of information in genomic prediction is reconstruction of relationship and noting that this marker-based reconstruction of relationships is shared by the three traits, we would have expected the same result for all the three traits. Because we see differences in behaviour between the three traits, these effects cannot be due to reconstruction of relationships but there must be differences between the traits in their genetic basis and in the LD relationship of QTL with markers versus haplotypes used as predictors. The fact that fewer non-zero effects are needed (higher π) for protein is consistent with the assumption that fewer factors influence protein yield than mastitis and fertility. Therefore, a relatively low number of haplotypes is sufficient to describe the inheritance of these factors for protein yield.

More covariates in the prediction models increase the computing times. With GBLUP, more time was needed to construct the **G** matrix with the haplotype-based prediction (20 min) than with the individual marker-based prediction (5 min), but the time required to obtain predictions was similar for the two prediction methods (~40 min) since the dimension of the **G** matrix was the same. For the Bayesian models, however, time to obtain predictions increased from 32 min when using individual markers to 3 hours and 20 min when using haplotypes.

## Conclusions

Accuracy of genomic prediction can be slightly improved and bias of prediction can be reduced by using haplotypes based on local genealogy information. The proportion of covariates with zero effects (π) has a large influence on the accuracy of genomic prediction when using Bayesian mixture models but haplotype-based prediction is less sensitive to π than individual marker-based prediction.

## Competing interests

The authors declare that they have no competing interests.

## Authors’ contributions

VE performed the statistical analysis and wrote the manuscript. RLF and GS provided the software, helped with the analysis and added valuable comments. RLF and BG conceived the study, made substantial contributions for interpretation of results and revised the manuscript. MSL, GS and BG coordinated the project. All authors read and approved the manuscript.
